# Results of Gastro-Jejunostomy

**Published:** 1905-09-02

**Authors:** 


### RESULTS OF GASTRO-JEJUNOSTOMY.

The operation of gastro-jejunostomy is so much in vogue at the present time that there is a danger of over-estimating its value in some instances and of invoking its aid with undue haste, or with too little discrimination. Therefore it cannot be too strongly emphasised that gastro-jejunostomy is not a panacea for every chronic gastric complaint. Used in suitable cases the operation may arrest decline and place a chronic invalid in a position of physical equality with his fellows. But in other cases, it may be after a short period of apparent benefit, the patient who has submitted to gastro-jejunostomy may find himself in no way improved by the procedure; and it may be a difficult matter for the medical attendant in such a case to satisfy his patient as to the necessity of having undergone the dangers and inconveniences of the operation.

To everyone who is interested in the matter, and who wishes to become acquainted with the after results of gastro-jejunostomy, we commend an excellent paper by Mr. D'Arcy Power, which recently appeared in the *Clinical Journal.* Mr. Power has carefully noted the ultimate results in a number of cases of gastro-jejunostomy and his conclusions are, as might be expected, most instructive. He classifies the cases into three groups, according to the condition for which the operation had been performed—namely, ulcer of the stomach, non-malignant obstruction of the pylorus or duodenum, and cancer.

In the first class of cases the results may be described as only moderately good. The patients, as a rule, experienced considerable benefit at first, but this was only temporary, and as a rule there was a recurrence of symptoms on their return to every-day life and habits. Mr. Power believes that better results may be obtained in the future in these cases by occluding the pylorus at the time the anastomosis is performed, for there is evidence that the artificial opening between the stomach and the jejunum has a tendency to close so long as it is possible for the gastric contents to escape through the pylorus. But he states that since he has adopted this procedure sufficient time has not elapsed to allow of a definite opinion being formed, and at present, in cases of dyspepsia associated with chronic gastric ulcer he feels it is only justifiable to tell a patient that although he may expect some relief, and might even be cured, he must not build his hopes too high upon the outcome of an operation.

It is the second group of cases which offers the most satisfactory field for the surgeon, for the results of gastro-enterostomy for non-malignant stricture of the pylorus or duodenum are very favourable, and it is safe to assure the patient that operation in such a case will cure him or bring very substantial and permanent relief from his sufferings.

In the third group, comprising cancer of the stomach, the question of cure, of course, hardly arises. . The operation prolongs life and relieves pain, but more than this is not to be expected.

